# Safety and clinical outcomes of orelabrutinib, lenalidomide plus sintilimab for relapsed/refractory diffuse large B-cell lymphoma

**DOI:** 10.3389/fimmu.2025.1629224

**Published:** 2025-09-01

**Authors:** Lingling Wang, Yongfen Huang, Dan Xu, Xiaoyong Wang, Hailiang Chu, Chunling Wang, Hao Xu, Wei Sang, Yuexin Cheng, Yuqing Miao

**Affiliations:** ^1^ Department of Hematology, The First People’s Hospital of Yancheng, The Yancheng Clinical College of Xuzhou Medical University, Yancheng, China; ^2^ Department of Hematology, Funing People’s Hospital, Yancheng, China; ^3^ Department of Hematology, Dongtai People’s Hospital, Yancheng, China; ^4^ Department of Hematology, Bozhou Hospital Affiliated to Anhui Medical University, Bozhou, China; ^5^ Department of Hematology, The Affiliated Huaian No.1 People’s Hospital of Nanjing Medical University, Huaian, China; ^6^ Department of Hematology, Affiliated Hospital of Xuzhou Medical University, Xuzhou, China

**Keywords:** diffuse large B-cell lymphoma, relapsed/refractory, immunotherapy, Bruton tyrosine kinase inhibitors, anti-programmed cell death-1 monoclonal antibody

## Abstract

**Introduction:**

This study evaluated the safety and clinical outcomes of orelabrutinib, lenalidomide plus sintilimab in relapsed/refractory (R/R) diffuse large B-cell lymphoma (DLBCL).

**Methods:**

Thirty-four patients were given orelabrutinib 150 mg once daily, lenalidomide 25 mg once daily on days 1–10, and sintilimab 200 mg intravenously on day 1 of each 21-day cycle.

**Results:**

With a median follow-up of 9 months (95% CI, 8.3-9.6), 7 patients died. The 1-year progression-free survival (PFS) and overall survival (OS) were 41.9% and 77.8%, respectively. The median PFS was 6 months (95% CI, 3.4-8.6), and median OS was not reached. The median exposure time was 4 months, while the median time to first response was 2 months. The best objective response rate (ORR) was 58.8%, with a complete remission (CR) rate of 38.2%. Twenty-eight (82%) patients presented with treatment-related adverse events (TRAEs), and 7 (20.6%) patients developed grade 3 or higher TRAEs. The most common grade 1 TRAEs were neutropenia (64.7%), thrombopenia (44.1%), skin rash (32.4%), and fatigue (29.4%). Patients who responded to treatment had a higher proportion of PD1^+^CD8^+^ T cells, a lower percentage of CD8^+^ T cells, and a higher percentage of CD4^+^ T cells and lower C-reactive protein (CRP) levels at baseline. Cytokines such as IL-6, IL-8, and IL-10 levels were also substantially lowered in these patients.

**Discussion:**

Orelabrutinib, lenalidomide plus sintilimab demonstrated promising efficacy and a manageable safety profile in Chinese patients with R/R DLBCL.

## Introduction

DLBCL is a highly aggressive lymphoma characterized by significant heterogeneity in genomic alterations, morphologic manifestations, clinical features, treatment response, and prognosis (1, 2). Despite the R-CHOP (rituximab, cyclophosphamide, doxorubicin, vincristine, and prednisone) regimen significantly improving survival, approximately 40% of patients ultimately experienced relapse or refractory disease (3). Patients with R/R DLBCL generally had a worse prognosis, especially those unable to tolerate conventional chemotherapy (4). Bruton tyrosine kinase inhibitors (BTKi) were selective inhibitors that target B-cell receptor (BCR) signaling, effectively blocking NF-κB activation downstream of BCR signaling and thereby controlling the development of B-cell lymphoma. Orelabrutinib (5), a potent and highly selective novel BTKi, has demonstrated specificity for BTK targets. Lenalidomide, an oral immunomodulator drug, has shown direct antitumor activity and immunological effects. Through the inhibition of NF-κB signaling, lenalidomide induces cytotoxicity in activated B-cell-like (ABC) DLBCL cells. Additionally, when BTKi is blocked B-cell receptor signaling, a synergistic effect takes place. The expression of programmed cell death 1 (PD-1)/programmed cell death ligand (PD-L1) on malignant cells was identified as a key immune escape mechanism in multiple tumors. Molecular profiling revealed that features of the immune response, rather than the characteristics of the lymphoma itself, might predict the clinical outcome (6). According to recent research (7), immune checkpoint inhibitors that rescue T-cells with low activity may improve this immune response. Early-stage clinical studies have already shown that non-Hodgkin lymphoma (NHL) responds to inhibitors of PD-1. Even for indolent lymphomas, only a small percentage of individuals respond to treatment, and responses in DLBCL are uncommon (8). By promoting antigen release through chemotherapy-induced cytotoxic cell death, the combination of immune checkpoint inhibitors and chemotherapy has been shown to increase immune responses and improve the effectiveness of PD-1/PD-L1 blockade (9). Given these observations, we anticipated that orelabrutinib, lenalidomide, and sintilimab, a chemotherapy-free regimen, could be effective in R/R DLBCL. Therefore, we conducted a retrospective study to evaluate the safety and clinical outcomes of orelabrutinib, lenalidomide plus sintilimab for R/R DLBCL. We monitored T-lymphocyte subsets and cytokines in a subset of patients to explore potential markers that are conveniently applicable to the clinic and can predict treatment efficacy early.

## Methods

### Patients

Patients with R/R DLBCL who received at least one line of systemic chemotherapy and were not considered candidates for high-dose chemotherapy or hematopoietic stem cell transplantation (HSCT) were included in this study. The study comprised patients who received continuous therapy and follow-up between September 2019 and August 2024. Baseline clinical characteristics, including gender, age, lactate dehydrogenase (LDH), Ann Arbor stage, International Prognostic Index (IPI)/National Comprehensive Cancer Network-revised (NCCN)-IPI risk category, Hans classification, number of prior therapy lines, prior best response (CR, partial remission [PR], stable disease [SD], or progressive disease [PD]), and prior treatment regimes, were collected from all patients. The study was conducted according to the principles of the Helsinki Declaration. The Ethics Committee of the First People’s Hospital of Yancheng approved this study (Approval No. 2021-K032). All patients provided written informed consent (including treatment, data use, and publication). Follow-up data were obtained by reviewing outpatient and inpatient medical records, supplemented by telephone follow-ups for all patients until September 25, 2024.

### Treatment

Patients were given orelabrutinib 150 mg once daily, lenalidomide 25 mg once daily on days 1–10, and sintilimab 200 mg intravenously on day 1 of each 21-day cycle. If grade 3 or higher neutropenia and/or a fever and infection combination occurred during previous therapy, prophylactic pegylated granulocyte colony-stimulating factor (Peg-G-CSF) was administered. Topical glucocorticoids or oral antihistamines were used to treat grade 1 skin rashes until they cleared up. When the adverse reaction was lowered to grade 1, consider continuing the treatment for rashes of grade 2 or above. If angioedema, grade 4 rash, exfoliative or maculopapular rash, Stevens-Johnson syndrome, toxic epidermal necrolysis, or a drug reaction with eosinophilia and systemic symptoms were suspected, stop taking the medication immediately. Patients with pulmonary fibrosis, squamous cell carcinoma, chronic obstructive pulmonary disease (COPD), previous chest radiation, combination therapy, or an active lung infection would be identified early. When immune-associated pneumonia is present, glucocorticoid treatment would be started immediately. Immunosuppressive therapy was recommended to be administered to patients with grade 3/4 immune-related pneumonia if symptoms fail to improve after 48 hours of starting glucocorticoid therapy. The regimen would be ceased when the patient’s disease progressed or the TRAEs proved unacceptable. Seventeen patients underwent peripheral blood sampling before treatment and within two months of receiving treatment. Flow cytometry was used to detect T lymphocyte subsets and cytokines.

### Outcomes and assessments

Baseline evaluations for all patients included computed tomography/positron emission tomography-computed tomography (CT/PET-CT) and bone marrow aspiration/biopsy. Response assessments were conducted according to the Lugano staging criteria every 4 cycles. Follow-up was performed every 3 months. The ORR was defined as the proportion of patients who achieved a CR or PR to treatment. Safety assessments include TRAEs, physical examination, laboratory tests (white blood cells, hemoglobin, platelet count, liver function, kidney function, coagulation panel), T lymphocyte subsets analysis, and cytokines analysis.

### Statistical analysis

SPSS version 26 was applied for statistical analysis. Descriptive statistical methods were used to summarize the characteristics of the patients. Medians were calculated to report statistical values. PFS was defined as the time from therapy beginning to disease progression, relapse, death, or last follow-up. Cox’s proportional hazards model was used to perform multivariate analysis. A significant difference was defined as a two-sided *P* value <0.05.

## Results

### Patients’ characteristics

Between September 2021 and August 2024, 34 patients who received continuous therapy and follow-up were included. Baseline clinical characteristics are detailed in **Table 1**. The median age was 59 years (range, 30–83 years), 58.8% (n=20) were males, 50% (n=17) were older than 60 years, and 58.8% (n=20) had B-symptoms. Non-germinal center B-cell like (non-GCB) subtype for immunohistochemistry (IHC) accounted for 91.2% (n=31), 55.9% (n=19) presented with Ann Arbor stages III-IV, 17.6% (n=6) had more than two prior lines of therapy, 85.3% (n=29) were in the state of relapse, and 8.8% (n=3) patients had undergone HSCT.

**Table 1:** Clinical characteristics.

Characteristics	
Gender, n (%)	
Male	14 (41.2)
Female	20 (58.8)
Age, n (%)	
< 60	17 (50.0)
≥ 60	17 (50.0)
B symptoms, n (%)	20 (58.8)
IPI risk category, n (%)	
Low-risk	7 (20.6)
Intermediate-risk	22 (64.7)
High-risk	5 (14.7)
NCCN-IPI risk category, n (%)	
Low-risk	6 (17.6)
Low-intermediate-risk	13 (38.2)
High-intermediate-risk	14 (41.2)
High-risk	1 (2.9)
LDH level ≥ 250 U/L, n (%)	15 (44.1)
Hans classification, n (%)	
GCB	3 (8.8)
Non-GCB	31 (91.2)
Ann Arbor Stage, n (%)	
I-II	15 (44.1)
III-IV	19 (55.9)
Number of prior therapy lines, n (%)	
1	17 (50.0)
2	11 (32.4)
3	3 (8.8)
4	2 (5.9)
5	1 (2.9)
Disease status, n (%)	
Refractory	5 (14.7)
Relapsed	29 (85.3)
Prior treatment regimes, n (%)	
R-CHOP	34 (55.7)
Gemox	16 (26.2)
Hyper-CVAD	1 (1.6)
GDP	6 (9.8)
EPOCH	4 (6.5)
Prior best response, n (%)	
CR	7 (20.6)
PR	26 (76.5)
SD	1 (2.9)
Prior auto-HSCT	3 (8.8)
Outcome (Survival), n (%)	27 (79.4)
Best response, n (%)	
CR	13 (38.2)
PR	7 (20.6)
SD	10 (29.4)
PD	4 (11.8)

CR, complete remission; EPOCH, etoposide, prednisone, vincristine (Oncovin), cyclophosphamide and doxorubicin; GCB, Germinal Center B-cell; GDP, gemcitabine, dexamethasone, cisplatin; Gemox: gemcitabine, oxaliplatin; HSCT, hematopoietic stem cell transplantation; Hyper-CVAD, hyper fractionated cyclophosphamide, vincristine, doxorubicin, and dexamethasone; IPI, International Prognostic Index; LDH, lactic dehydrogenase; NCCN, National Comprehensive Cancer Network; PD, progressive disease; PR, partial remission; R-CHOP, rituximab, cyclophosphamide, doxorubicin, vincristine, and prednisone; SD, stable disease.

### Efficacy

**Figure 1:**
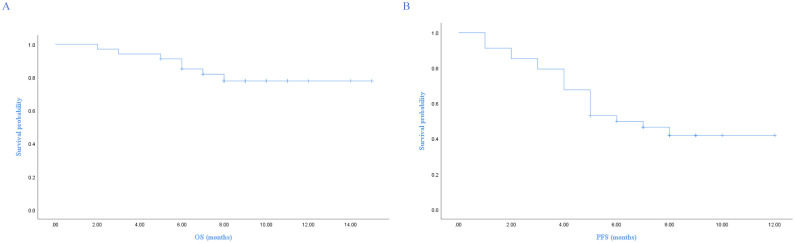
Kaplan-Meier survival curves of OS and PFS. **(A)** Overall survival, **(B)** Progression-free survival.

**Figure 2:**
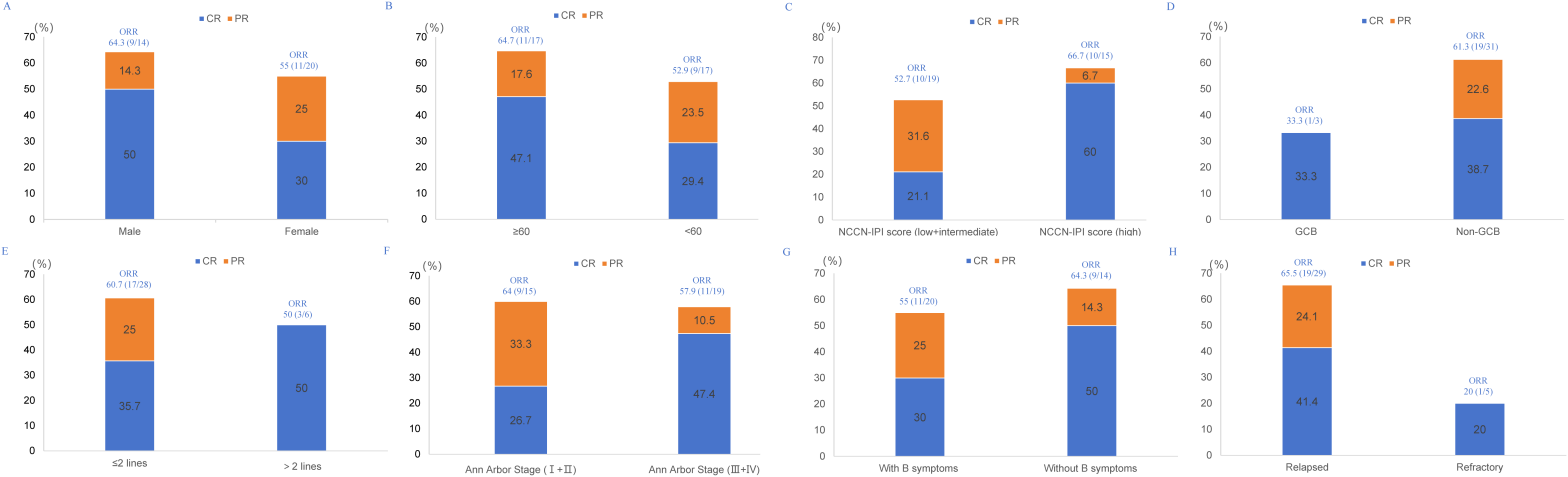
Treatment response in each subgroup. **(A)** gender, **(B)** age, **(C)** NCCN-IPI risk category, **(D)** Hans classification, **(E)** number of prior therapy lines, **(F)** Ann Arbor Stage, **(G)** B symptoms, **(H)** disease status.

With a median follow-up of 9 months (95% CI, 8.3-9.6), 7 patients died. The 1-year PFS and OS were 41.9% and 77.8%, respectively. The median PFS was 6 months (95% CI, 3.4-8.6), and median OS was not reached. The Kaplan-Meier survival curves for PFS and OS are displayed in **Figures 1A, B**. The median exposure time was 4 months (range, 1–8 months), while the median time to first response was 2 months (range, 1-3.5 months). Based on the best response, 38.2% (n=13) achieved CR, 20.6% (n=7) achieved PR, 29.4% (n=10) were SD, and 11.8% (n=4) were PD (**Table 1**). The best ORR was 58.8% (95% CI, 41.4%-76.3%). **Figure 2** presents the treatment response in each subgroup. In comparison to the GCB group, the non-GCB group (**Figure 2D**) indicated a higher remission rate (61.3% vs. 33.3%). Patients who were severely pretreated (≥2 lines vs. <2 lines, 50.0% vs. 60.7%) (**Figure 2E**) and refractory (refractory vs. relapsed, 20% vs. 65.5%) (**Figure 2H**) had a lower ORR trend. Univariate and multivariate Cox analyses were conducted for PFS, as detailed in **Table 2**. However, none of these variables constituted independent factors for PFS.

**Table 2:** Univariate and multivariate Cox regression model for PFS.

Variable	Univariate analysis			Multivariate analysis		
	HR	95% CI	P-value	HR	95% CI	P-value
Gender (Male/Female)	1.039	0.232-4.647	0.960			
Age (≥ 60/< 60)	2.365	0.458-12.202	0.304	0.214	0.025-1.841	0.16
Disease status (Relapsed/Refractory)	1.066	0.128-8.861	0.953			
Ann Arbor Stage (I+II/III+IV)	0.488	0.095-2.516	0.391			
NCCN-IPI score (high/low+intermediate)	1.195	0.267-5.349	0.816			
Hans classification (non-GCB/GCB)	3.666	0.432-31.109	0.234	0.067	0.004-1.241	0.069
B symptoms (No/Yes)	0.019	0.000-8.797	0.205			
Number of prior therapy lines(> 2/≤ 2)	1.332	0.160-11.070	0.791			
Best Response (CR+PR/PD+SD)	0.342	0.075-1.546	0.163	3.622	0.751-17.457	0.109

CR, complete remission; GCB, Germinal Center B-cell; HR, hazard ratio; IPI, International Prognostic Index; NCCN, National Comprehensive Cancer Network; PD, progressive disease; PFS, progression-free survival; PR, partial remission; SD, stable disease.

### T lymphocyte subsets and cytokines analysis

**Figure 3:**
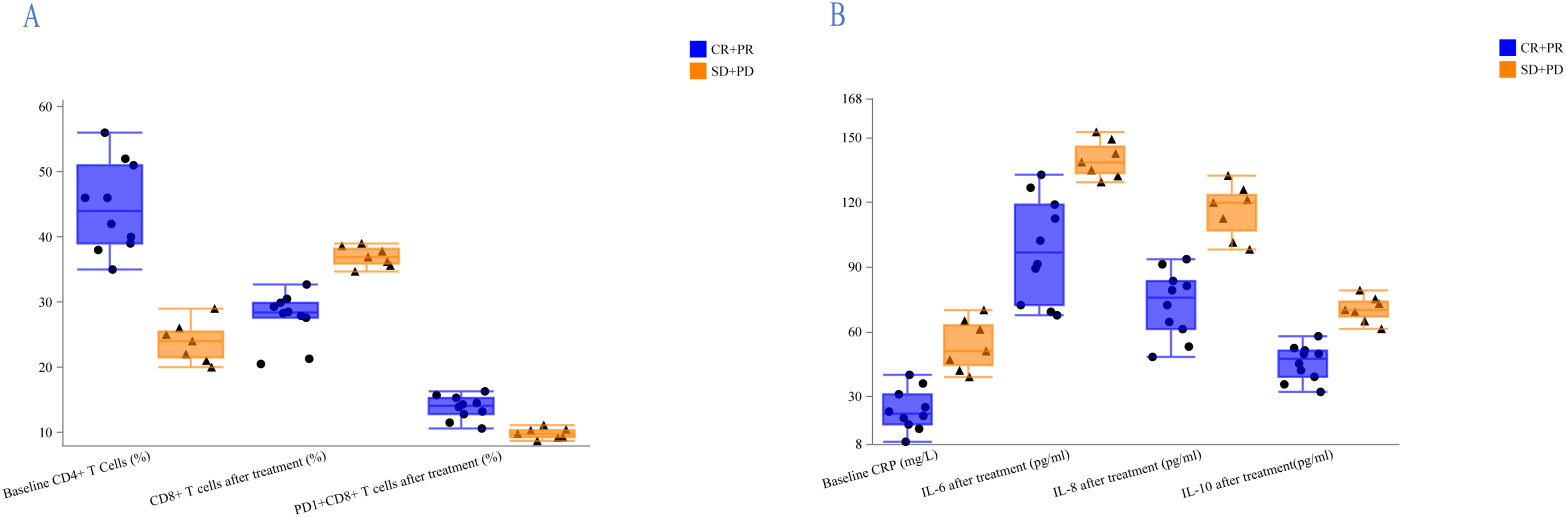
Analysis of lymphocyte subsets and cytokines in the CR+PR and SD+PD groups. **(A)** Patients who responded to treatment had a higher proportion of PD1^+^CD8^+^ T cells, a lower percentage of CD8^+^ T cells, and a higher percentage of CD4^+^ T cells at baseline. **(B)** Treatment-responsive patients exhibited lower CRP levels at baseline and lower levels of IL-6, IL-8, and IL-10 following treatment.

T-lymphocyte subsets and cytokines were tracked in 17 patients (CR, n=7; PR, n=3; PD, n=4; SD, n=3) both at baseline and during the first two months of treatment. T-lymphocyte subsets and cytokines across different treatment response modes are shown in **Figure 3**. For groups of CR+PR and SD+PD, the median PD1^+^CD8^+^ T cells after therapy were 14.1% and 9.8%, respectively, and median CD8^+^ T cells were 28.4% and 36.9%, respectively, while at baseline the median CD4^+^ T cells were 44.0% and 24.0%, respectively (**Figure 3A**). The median CRP at baseline for the CR+PR and SD+PD groups was 22.0 mg/L and 51.0 mg/L, respectively. Following therapy, the median levels of IL-6, IL-8, and IL-10 were 96.75 pg/mL and 138.5 pg/mL, 75.75 pg/mL and 119.8 pg/mL, and 47.45 pg/mL and 70.1 pg/mL, respectively (**Figure 3B**). There was a trend of a higher proportion of PD1^+^CD8^+^ T cells, a lower percentage of CD8^+^ T cells, and lower levels of IL-6, IL-8, and IL-10 in patients who responded to treatment.

### Safety

**Figure 4:**
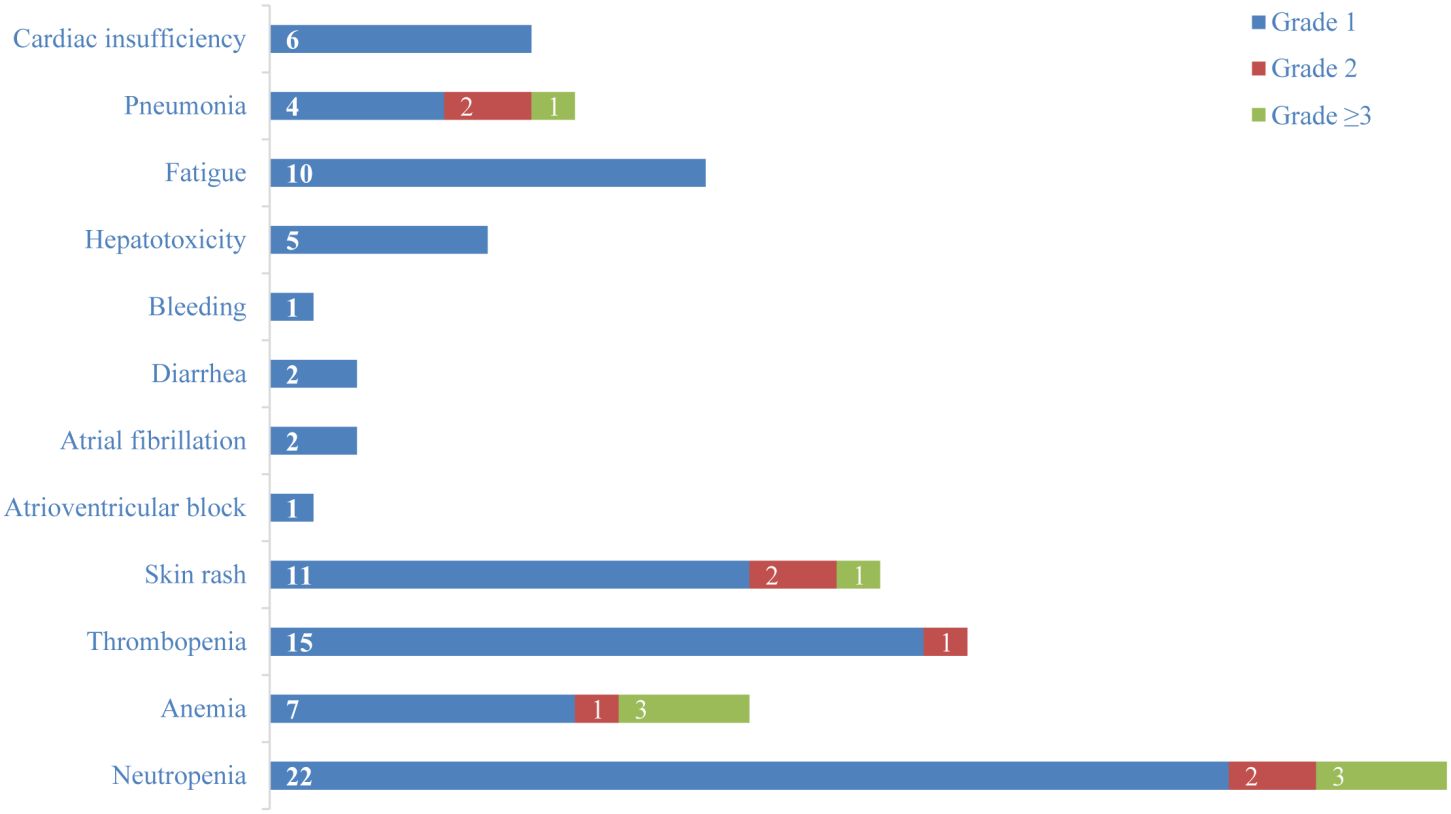
Adverse events.

82% (n=28) of patients presented with TRAEs, and 20.6% (n=7) of patients developed grade 3 or higher TRAEs (**Figure 4**). Among grade 1 hematologic adverse events, neutropenia (64.7%, n=22) and thrombopenia (44.1%, n=15) were the most frequent. Skin rash (32.4%, n=11) and fatigue (29.4%, n=10) were the most common grade 1 non-hematologic TRAEs. 23.5% (n=8) of patients developed infections, with pneumonia (20.6%, n=7) being the most frequent. In addition, 17.6% (n=6) of patients developed grade 1 cardiac insufficiency, and patients were able to improve their symptoms after supportive therapy. One patient developed an atrioventricular block, and two developed atrial fibrillation. Immune-related adverse events (irAEs) such as increased creatinine, hypothyroidism, myocarditis, pancreatitis, and encephalitis were not observed. By September 25, 2024, 20.6% (n=7) of patients died, but none of them were related to treatment.

## Discussion

BTKi can selectively inhibit BTK activity, and it intervenes in B-cell development by regulating the BCR signaling pathway to control the further development of various B-cell malignant diseases, and regulates the FcγR signaling pathway to treat autoimmune diseases. Currently, there are 5 types of BTKi approved and marketed: ibrutinib, acalabrutinib, zanubrutinib, tirabrutinib, and orelabrutinib. Orelabrutinib was a novel, potent, and irreversible BTKi with higher kinase selectivity and fewer off-target effects, offering significant advantages in terms of toxicity and safety (10, 11). Unlike traditional BTKi, which might affect other kinases (such as TEC, BMX, ITK, EGFR, etc.) due to sequence similarity and shared cysteine residue at the adenosine triphosphate binding site, orelabrutinib minimizes these off-target interactions. The lack of great selectivity in conventional BTKi raises the possibility of off-target effects that could result in adverse reactions. By strengthening its coordination with the BTK active site, orelabrutinib’s molecular structure has been modified to improve BTK-specific inhibition. This optimization not only enhanced efficacy but also markedly reduced off-target side effects, as evidenced by preliminary research (12).

Orelabrutinib-based regimens have shown encouraging results for DLBCL. Fang Jun et al. carried out a retrospective study (13). Nineteen DLBCL patients (17 newly diagnosed, 2 relapsed) were treated with orelabrutinib-based combination treatments plus R-CHOP, R-GDP (rituximab, dexamethasone, gemcitabine, and cisplatin), Rituximab plus methotrexate (MTX), or MA (MTX and cytosine arabinoside). The ORR and CR rates were 89.5% and 73.7%, respectively. Orelabrutinib, along with R-CHOP, was also found to be efficacious in newly diagnosed *MYD88^mut^* and/or *CD79B^mut^* DLBCL. According to the study, the best ORR, CR, and 1-year PFS rates were 100%, 94.4%, and 88.9%, respectively (14). Orelabrutinib has also shown promise in treating R/R central nervous system lymphoma (CNSL) patients (15). Fourteen individuals with primary or secondary DLBCL of the central nervous system (CNS) were included in a retrospective study. Each patient was given orelabrutinib, thiotepa, and high-dose MTX (HD-MTX) with or without rituximab (MTO ± R). The study’s findings showed a 92.3% ORR and a CR rate of 69.2%. For primary CNSL, the ORR was 88.9% and the CR rate was 55.6%. The ORR and CR rate for R/R CNSL were 91.7% and 66.7%, respectively. The median OS has not yet been reached, while the median PFS was 11.3 months. The 1-year PFS and OS rates were 60% and 70%, respectively. Another study included 37 patients with R/R CNSL who received orelabrutinib+HD-MTX-based regimens (16). The ORR was 89.2%, with a CR rate of 51.4%. The median PFS was 7.0 months. For individuals with R/R CNSL, the combination of orelabrutinib with chemotherapy provides a novel therapeutic option. These findings indicate that orelabrutinib is an effective treatment both in newly diagnosed and R/R DLBCL.

Mark Roschewski et al. (17) evaluated the efficacy of acalabrutinib with lenalidomide and rituximab (R2A) in patients with R/R B-cell NHL (ClinicalTrials.gov registration number: NCT04094142). There were 61 R/R DLBCL patients in the study. The findings revealed a median duration of response (DoR) of 12.9 months, an ORR of 54.5%, a CR rate of 31.8%, and 1-year PFS and OS rates of 33.1% and 67.5%, respectively. Additionally, 42 patients had their relevant biomarkers evaluated. Those with *MYD88* mutations, NF-κB activation subtypes, and higher BTK expression by IHC showed a good response to treatment. Our findings indicated that the 1-year PFS and OS were 41.9% and 77.8%, respectively. The median PFS was 6 months, and median OS was not reached. The ORR was 58.8%, with a CR rate of 38.2%. Finding safe and efficient salvage chemotherapy regimens is crucial given the difficulties presented by R/R DLBCL, particularly for frail patients who decline ASCT and CAR-T treatment. Our retrospective study provided a basis for further research and preliminary evidence of the effectiveness of this chemotherapy-free treatment in R/R DLBCL, which is in line with earlier findings.

Lenalidomide and ibrutinib together have a synthetic lethality against DLBCL (18). In the Smart Start study (19), Westin et al. showed that a chemotherapy-free combination of rituximab, ibrutinib, and lenalidomide was successful in treating patients with newly diagnosed non-GCB DLBCL. In patients with R/R DLBCL, especially non-GCB DLBCL, the combination of ibrutinib, lenalidomide, and rituximab showed encouraging activity, according to Goy et al (20).

The non-GCB immunophenotype and worse therapy outcomes have been linked to DLBCL expression of PD-L1. Although anti-PD-1 monoclonal antibody (mAb) alone has poor efficacy in R/R DLBCL, Ajay K Gopal et al. (21) conducted a study on the treatment of newly diagnosed DLBCL with pembrolizumab in combination with R-CHOP, taking into account the relatively intact immune function of first-line treatment patients, the tumor microenvironment where tumor cells and T cells coexist, and the potential synergistic mechanism of anti-PD-1 mAb in combination with R-CHOP. The study included thirty patients in total. The study revealed ORR, CR rate, and 2-year PFS rates of 90%, 77%, and 83%, respectively. In R/R DLBCL, anti-PD-1 mAb has also shown excellent effectiveness. Huang et al. investigated the application of anti-PD-1 mAb in conjunction with ICE (ifosfamide, carboplatin, and etoposide). The study included 67 patients, with a median follow-up time of 24.7 months. The ORR, CR rate, and 2-year PFS rate were 62.7%, 43.3%, and 41.1%, respectively (22).

Jerome Ritz et al. (23) assessed the kinetics of peripheral blood immune cell recovery following autologous stem cell transplantation (ASCT) for participants receiving pembrolizumab maintenance versus those of a contemporaneous control cohort of comparable patients undergoing ASCT without pembrolizumab maintenance to shed light on the effect of pembrolizumab on immune reconstitution. The purpose of this study was to determine potential biomarkers of efficacy and irAEs, as well as to describe the effects of post-ASCT pembrolizumab maintenance therapy on immunological reconstitution in patients with R/R DLBCL. The study found that pembrolizumab maintenance therapy post-ASCT was linked to a significant decrease in PD-1^+^ T cells that lasted for 6 to 12 months following the end of pembrolizumab medication, as well as an increase in circulating dendritic cells that lasted the course of pembrolizumab treatment. The recovery of any T cell subgroup was unaffected by pembrolizumab maintenance, even though T cells play a crucial role in mediating the effects of PD-1 blockage. A greater baseline CD4^+^ terminal effector memory cell count (defined as CD3^+^CD4^+^CD45RA^+^CD62L^-^) was linked to a lower PFS in an exploratory study, but only in patients who were maintained on pembrolizumab (*P*=0.003). Patients with R/R DLBCL who underwent anti-PD-1 mAb and rituximab regimens as salvage therapy were gathered for a retrospective study (24). According to the study, non-response patients had significantly higher alterations in *TP53* (*p*=0.015) and *CREBBP* (*p*=0.029). The OS was longer for patients with PD-L1 CPS > 5 than for those with PD-L1 CPS < 5.

Responses to regimens comprising anti-PD-1 mAb vary among patients with R/R DLBCL. It’s questionable what the processes and predictive biomarkers for this regimen’s effectiveness are. According to earlier research, there are still a lot of obstacles in clinical practice, even if tumor tissue-based indicators can be useful in assessing whether a patient can benefit. First, tumor biopsies are frequently intrusive procedures, and patient state and tumor accessibility significantly restrict the utilization of biopsies to obtain tissue samples from patients. Repeated tissue biopsies may postpone cancer therapy and raise the risk of procedure-related problems. Furthermore, a local immune response in a metastatic site could not accurately reflect a patient’s systemic anticancer immunity because of tumor heterogeneity. More thorough immunoassays can now be performed on peripheral blood thanks to the development of high-throughput analytic tools. During cancer immunotherapy, the use of blood-based immune biomarkers can make up for the previously described drawbacks of tissue-based immune biomarkers because peripheral blood samples are readily available, less invasive, and repeatable.

Since most T cells migrate to tumor tissue, a decrease in CD8^+^ T lymphocytes is related to durable clinical benefit. The T cell subset linked to cancer immunity is extremely varied and not tumor-specific, despite the fact that the total amount of circulating CD8^+^ T cells in peripheral blood is a sign of the overall immune status. During the tumor immune cycle, T cell immunological tolerance and depletion are regulated by the interaction between PD-1 and PD-L1. When PD-1/L1 blockage revitalizes PD1^+^CD8^+^ T cells and triggers their activation and proliferation, these proliferative T cells show an effector phenotype. While patients with delayed or no response to PD-1-targeted therapy usually experience disease progression, those who exhibit early proliferative T-cells may benefit from PD-1-targeted therapy. Thus, the early appearance of proliferating PD-1^+^CD8^+^ T cells after immune checkpoint inhibitor therapy may serve as a predictor of clinical response. We conducted an initial investigation of patients’ immunological function in this study. We tracked CD8^+^ T cells, PD1^+^CD8^+^ T cells, and cytokines such as CRP, IL-6, IL-8, and IL-10 in 17 patients. The findings demonstrated that patients who responded to treatment had a higher proportion of PD1^+^CD8^+^ T cells, and a lower percentage of CD8^+^ T cells, IL-6, IL-8, and IL-10. But which time point is most suitable for recognizing the dynamic changes of PD-1^+^CD8^+^ T cells requires more research. It must be acknowledged, however, that our monitoring had shortcomings, and the aforementioned indicators were insufficient to accurately represent the clinical benefit and therapeutic response of patients following immunotherapy. Other shortcomings include the lack of gene expression profiling-based typing (e.g., *MYD88^mut^*/*CD79B^mut^* ^)^. Genotyping can be clarified by genetic testing; for example, patients with the MCD subtype are more responsive to BTKi, and following BTKi-based therapy, these patients exhibit good remission and survival benefits. Furthermore, it is important to note that the follow-up time is still short and that the number of cases receiving this treatment is still limited. Following treatment, only a small percentage of patients had their T lymphocyte subsets and cytokines monitored. This regimen’s long-term efficacy has to be further confirmed by multi-center cooperation and a larger number of cases. In general, our preliminary analysis of the orelabrutinib-based chemotherapy-free therapy sets the stage for more extensive research. Multi-color flow cytometry, mass cytometry, and next-generation sequencing (NGS) will be used in our following study to identify different immune cell subsets in patients who have shown a sustained response to treatment. The biomarkers include, but are not limited to, TCR clonality and diversity of PD1^+^CD8^+^ T cells. We also intend to investigate which time point for circulating immune cell samples best predicts an early response to treatment.

## Conclusion

This study evaluated the safety and clinical outcomes of a chemotherapy-free regimen combining the novel BTKi (orelabrutinib), sintilimab, and lenalidomide in R/R DLBCL. The triple-drug combination, targeting multiple mechanisms, achieved anti-tumor efficacy and showed favorable tolerability in Chinese patients with R/R DLBCL.

## Data Availability

The original contributions presented in the study are included in the article/supplementary material. Further inquiries can be directed to the corresponding author.
